# Gestational Age Patterns of Fetal and Neonatal Mortality in Europe: Results from the Euro-Peristat Project

**DOI:** 10.1371/journal.pone.0024727

**Published:** 2011-11-16

**Authors:** Ashna D. Mohangoo, Simone E. Buitendijk, Katarzyna Szamotulska, Jim Chalmers, Lorentz M. Irgens, Francisco Bolumar, Jan G. Nijhuis, Jennifer Zeitlin, the Euro-Peristat Scientific Committee

**Affiliations:** 1 Department Child Health, TNO Netherlands Organization for Applied Scientific Research, Leiden, The Netherlands; 2 Department of Epidemiology, National Research Institute of Mother and Child, Warsaw, Poland; 3 Information Services Division, NHS National Services Scotland, Edinburgh, Scotland; 4 Department of Public Health and Primary Health Care, University of Bergen and Medical Birth Registry of Norway, Norwegian Institute of Public Health, Norway; 5 Department of Public Health Sciences, University of Alcalá, Madrid, Spain; 6 Maastricht University Medical Center, GROW School for Oncology and Developmental Biology, Maastricht, The Netherlands; 7 INSERM, UMRS 953, Epidemiological Research Unit on Perinatal and Women's and Children's Health, Paris, France; 8 UPMC Univ Paris 06, Paris, France; The University of Adelaide, Australia

## Abstract

**Background:**

The first European Perinatal Health Report showed wide variability between European countries in fetal (2.6–9.1‰) and neonatal (1.6–5.7‰) mortality rates in 2004. We investigated gestational age patterns of fetal and neonatal mortality to improve our understanding of the differences between countries with low and high mortality.

**Methodology/Principal Findings:**

Data on 29 countries/regions participating in the Euro-Peristat project were analyzed. Most European countries had no limits for the registration of live births, but substantial variations in limits for registration of stillbirths before 28 weeks of gestation existed. Country rankings changed markedly after excluding deaths most likely to be affected by registration differences (22–23 weeks for neonatal mortality and 22–27 weeks for fetal mortality). Countries with high fetal mortality ≥28 weeks had on average higher proportions of fetal deaths at and near term (≥37 weeks), while proportions of fetal deaths at earlier gestational ages (28–31 and 32–36 weeks) were higher in low fetal mortality countries. Countries with high neonatal mortality rates ≥24 weeks, all new member states of the European Union, had high gestational age-specific neonatal mortality rates for all gestational-age subgroups; they also had high fetal mortality, as well as high early and late neonatal mortality. In contrast, other countries with similar levels of neonatal mortality had varying levels of fetal mortality, and among these countries early and late neonatal mortality were negatively correlated.

**Conclusions:**

For valid European comparisons, all countries should register births and deaths from at least 22 weeks of gestation and should be able to distinguish late terminations of pregnancy from stillbirths. After excluding deaths most likely to be influenced by existing registration differences, important variations in both levels and patterns of fetal and neonatal mortality rates were found. These disparities raise questions for future research about the effectiveness of medical policies and care in European countries.

## Introduction

In December 2008, the Euro-Peristat network produced the first European Perinatal Health Report (EPHR) with data from 25 participating EU member states and Norway [1]. This report showed great variations between European countries in fetal and neonatal mortality rates in 2004 [1]–[3]. The highest mortality rates were approximately 3.5 times higher than the lowest. Fetal mortality rates ranged from 2.6 to 9.1 per 1000 total births, and neonatal mortality rates from 1.6 to 5.7 per 1000 live births. The EPHR also showed that, despite efforts of the World Health Organization (WHO) to promote the use of common inclusion criteria, there were still substantial differences in limits for registration of live and stillbirths in Europe in 2004, especially stillbirths [4]. These registration differences could be one explanation for the observed variability between countries.

Preterm birth is an important risk factor for mortality during the perinatal period and a key to understanding the etiology of both fetal and neonatal deaths. One of the recommendations of the Euro-Peristat project was therefore to collect and present data on fetal and neonatal mortality by gestational age to allow for exclusion of gestational age groups when differences in registration are most marked and to permit more meaningful analysis of variations between countries by comparing gestational age-specific mortality rates. Differences in health care policies and practices may contribute to the variation in observed fetal and neonatal mortality rates by gestational age, including, for instance, policies related to screening and terminations for congenital anomalies [5]–[6]. In countries where terminations are not legal, some babies with severe congenital anomalies probably die at later gestational ages either during pregnancy or during the neonatal period. In other countries, these pregnancies are more likely to be terminated at earlier gestational ages. Furthermore, prevention strategies for reducing mortality may differ for very preterm versus at and near term births between European countries. For instance, programs to improve the regionalization of perinatal care can contribute to reduced mortality in the very preterm population [7].

The aim of this study was to analyze gestational age-related differences in fetal and neonatal mortality between countries in order to assess which part of inter-country variation is due to variations in registration of births and deaths and which part is due to real differences in health and quality of care. We also sought to improve our understanding of differences between low versus high mortality countries by identifying patterns of mortality by gestational age. The following research questions will be addressed: (i) How did preterm deaths, and in particular early preterm deaths, contribute to differences in the variability of fetal and neonatal mortality rates? (ii) Were absolute mortality rates associated with a specific pattern? That is, did countries with low mortality rates have higher proportions of preterm deaths (which might be considered less preventable) and did high mortality countries have higher proportions of at and near term deaths? (iii) How did the timing of mortality (fetal/early neonatal/late neonatal) differ in countries with high versus low mortality?

## Methods

This study was embedded within the Euro-Peristat project, which developed a list of valid and reliable indicators for monitoring and evaluating perinatal health in the European Union [8]. Twenty-five EU member states and Norway participated. Detailed information on the design and methods of the Euro-Peristat project is available elsewhere [4], [9]–[10].

Data collection was coordinated in the Netherlands. Data about 29 countries/regions for the year 2004 were analyzed: Austria (AT), Belgium (BE): regions Brussels (BE.BR) and Flanders (BE.FL), Cyprus (CY), Czech Republic (CZ), Denmark (DK), Estonia (EE), Finland (FI), France (FR), Germany (DE), Greece (GR), Hungary (HU), Ireland (IE), Italy (IT), Latvia (LV), Lithuania (LT), Luxembourg (LU), Malta (MT), the Netherlands (NL), Norway (NO), Poland (PL), Portugal (PT), Slovenia (SI), Slovakia (SK), Spain or the Valencia region of Spain (ES), Sweden (SE), and the United Kingdom (UK): England and Wales combined (UK.EW), Northern Ireland (UK.NI), and Scotland (UK.SC).

### Euro-Peristat definitions

Within Euro-Peristat the fetal mortality rate is defined as the number of deaths before or during birth at or after 22 completed weeks of gestation in a given year per 1000 live and stillbirths in the same year. The neonatal mortality rate is defined as the number of deaths during the neonatal period (day 0 to 27) at or after 22 completed weeks of gestation in a given year expressed per 1000 live births in the same year. Early preterm deaths were defined as deaths that occurred at 22–27 weeks of gestation, and at and near term deaths as deaths at 37 weeks and above.

### Availability of fetal and neonatal mortality

If participating countries/regions were unable to provide numbers on fetal and neonatal mortality according to the Euro-Peristat definition, the local definition was used. Cyprus provided no data on fetal mortality, and the data from Greece and Italy were for 2003. Information on fetal deaths with and without TOP was collected afterwards, when it appeared that TOP were not systematically included as fetal deaths.

All countries/regions provided data on neonatal mortality. Ireland provided data only on early neonatal mortality and Germany only on early neonatal mortality by gestational age. Cyprus, the Czech Republic, Greece, Hungary, and Italy provided no data on neonatal mortality by gestational age.

Spanish data by gestational age came only from the Valencia region. Data from France by gestational age was based on several sources: a one-week national perinatal survey that was conducted in October 2003, vital registration, and neonatal death certificates. Data on the gestational age distribution for live births from England and Wales related to 2005, since these data were not available at a national level before. Detailed information on the data sources used is presented in Table S1.

### Statistical analysis

The annual number of births ranged from 3902 to 774 870. France, Germany, England and Wales, and Italy were among the countries with more than 500 000 births, while Malta, Luxembourg, and Cyprus had less than 10 000. We therefore calculated confidence intervals using the binomial distribution to deal with statistical variation of observed mortality rates between countries. Rates were not calculated if there were fewer than 10 births. Rates based on fewer than 10 deaths are noted in the tables.

Low and high mortality countries were defined by choosing the 25^th^ and 75^th^ percentiles respectively as cut-off levels. Differences in the proportions of fetal/neonatal deaths between low versus high mortality countries were tested with the χ^2^-test. We used the non-parametric Spearman test to assess correlations and thus minimize the effects of outliers. Spearman's rho (ρ) was used to interpret the strength of correlations. All analyses were performed with SPSS version 17.0 for Windows (SPSS Inc, Chicago, IL, USA).

## Results

### Registration of live and stillbirths

Table 1 shows that most European countries had no limits for registration of live births in 2004, but that the legal limits for registration of stillbirths varied substantially. In some countries stillbirths weighing less than 500 grams were not registered, while other countries had a legal gestational age limit of 24 or even 28 weeks. Because of this Hungary and Sweden could not adhere to the Euro-Peristat definition of 22 completed weeks of gestation for stillbirths in 2004. Voluntary notification of late fetal deaths at 22–23 weeks existed in the United Kingdom and Portugal and therefore Portugal, Northern Ireland, and Scotland were able to include these deaths in their data. Italy and Luxembourg had a legal limit for registration of 180 days of pregnancy, but late fetal deaths starting at 22 weeks were available in the register of spontaneous abortions and were included. Table 1 also indicates that most countries did not include TOP as fetal deaths. Exceptions were France, the Netherlands and Scotland. Elsewhere TOP were registered separately and not included in national mortality statistics.

**Table 1:** Criteria for registration of live births and stillbirths and inclusion of terminations of pregnancy in Europe in 2004.

	Live births	Stillbirths	TOP included	TOP included in a separate data system
Austria	No limit	≥500 grams	No	No
BE: Brussels	No limit	≥22 weeks or ≥500 grams	No	No
BE: Flanders	No limit	≥500 grams	No	No
Cyprus	No limit	No data available	No data available	
Czech Republic	≥500 grams or any birth weight surviving the first 24 hours	≥22 weeks	No	Yes
Denmark	No limit	≥22 weeks	No	Yes
Estonia	No limit	≥22 weeks or ≥500 grams	No	Yes
Finland	No limit	≥22 weeks or ≥500 grams	No	Yes
France	≥22 weeks or ≥500 grams	≥22 weeks or ≥500 grams	Yes	No
Germany	No limit	≥500 grams	No	Yes
Greece	No limit	legal limit of ≥28 weeks	No	No
Hungary	No limit	≥24 weeks or ≥500 grams	No	Yes
Ireland	No limit	≥24 weeks or ≥500 grams	TOP is not legal and not performed	
Italy	No limit	180 days	No	Yes
Latvia	No limit	≥22 weeks	No	Yes
Lithuania	≥22 weeks	≥22 weeks	No	Yes
Luxembourg	No limit	180 days	No	No
Malta	No limit	≥22 weeks or ≥500 grams	TOP is illegal and not performed	
The Netherlands	≥22 weeks or ≥500 grams	≥24 weeks for civil registration≥16 weeks for perinatal registry	Yes	Yes
Norway	≥12 weeks	≥12 weeks	No	Yes
Poland	≥500 grams	≥500 grams	No	Yes
Portugal	No limit	≥24 weeks	No	No
Slovakia	No limit	≥22 weeks or ≥500 grams	No	Yes
Slovenia	No limit	≥500 grams	No	Yes
Spain	No limit	≥26 weeks (national)≥22 weeks (region Valencia)	No	Yes
Sweden	No limit	≥28 weeks	No	Yes
UK: England and Wales	No limit	legal limit of ≥24 weeks voluntary notification at 22–23 weeks	Yes	Yes
UK: Northern Ireland	No limit	legal limit of ≥24 weeks voluntary notification at 22–23 weeks	TOP is not legal*	
UK: Scotland	No limit	legal limit of ≥24 weeks voluntary notification at 22–23 weeks	Yes	Yes

*The legislation which legalised abortion in the rest of the United Kingdom does not cover Northern Ireland, but TOP are occasionally done there under case law.

### Variation in fetal and neonatal mortality rates

A large range was observed for overall fetal (2.6–9.1‰) and neonatal (1.6–5.7‰) mortality rates in the 28 participating countries/regions, as shown in Table 2. In these countries/regions, 25 360 fetal deaths and 4 733 268 births, and 14 212 neonatal deaths and 4 713 200 live births were registered. The proportion of fetal deaths represented by TOP varied between countries, as measured by supplemental data provided by a few countries (data not shown in table). If TOP were included as fetal deaths, the fetal mortality rate would have increased from 5.7‰ to 5.9‰ in England and Wales (3.3% were TOP), from 3.2‰ to 3.7‰ in Finland (16% were TOP), and from 5.4‰ to 6.9‰ in Italy (28% were TOP). After excluding TOP, the fetal mortality rate in Scotland would have declined from 6.7‰ to 6.6‰ (2.5% were TOP), and the Netherlands estimated that their fetal mortality rate would have declined from 7.0‰ to 6.8‰ after excluding TOP (2.9% were TOP).

**Table 2:** Fetal and neonatal mortality rates in Europe in 2004.

Country/region	Number of total births	Number of fetal deaths	Fetal Mortality Rates per 1000 total births	Number of live births	Number of neonatal deaths	Neonatal Mortality Rates per 1000 live births
Austria	79 229	295	3.7 [3.3–4.1]	78 934	215	2.7 [2.4–3.1]
BE: Brussels	16 288	88	5.4 [4.3–6.5]	16 200	51	3.4 [2.3–4.0]
BE: Flanders	60 921	249	4.1 [3.6–4.6]	60 672	146	2.4 [2.0–2.8]
Cyprus	NA	NA	NA	8 309	13	1.6 [0.7–2.4]
Czech Republic	98 051	387	3.9 [3.6–4.3]	97 671	224	2.3 [2.0–2.6]
Denmark	64 853	332	5.1 [4.6–5.7]	64 521	230	3.6 [3.1–4.0]
Estonia	14 053	63	4.5 [3.4–5.6]	13 990	59	**4.2 [3.1–5.3**]
Finland	57 759	190	3.3 [2.8–3.8]	57 569	141	2.4 [2.0–2.9]
France	774 870	7 054	**9.1 [8.9–9.3]**	767 816	1968	2.6 [2.5–2.7]
Germany	648 860	2 261	3.5 [3.3–3.6]	705 622	1892	2.7 [2.6–2.8]
Greece	104 858	503	4.8 [4.4–5.2]	104 355	282	2.7 [2.4–3.0]
*Hungary*	95 594	476	*5.0 [4.5–5.4]*	95 137	423	**4.4 [4.0–4.9]**
Ireland	62 400	334	5.4 [4.8–5.9]	62 066	NA	NA
Italy	542 003	2 937	5.4 [5.2–5.6]	539 066	1526	2.8 [2.7–3.0]
Latvia	20 492	137	**6.7 [5.6–7.8]**	20 355	116	**5.7 [4.7–6.7]**
Lithuania	29 633	153	5.2 [4.3–6.0]	29 480	136	**4.6 [3.8–5.4]**
Luxembourg	5 486	17	3.2 [1.6–4.6]	5 469	11	2.0 [0.8–3.2]
Malta	3 902	15	3.8 [1.9–5.8]	3 887	17	**4.4 [2.3–6.4]**
The Netherlands	182 279	1 273	**7.0 [6.6–7.4]**	181 006	631	3.5 [3.2–3.8]
Norway	57 368	257	4.5 [3.9–5.0]	57 111	118	2.1 [1.7–2.4]
Poland	358 440	1 743	4.9 [4.6–5.1]	356 697	1731	**4.9 [4.6–5.1]**
Portugal	109 778	422	3.8 [3.5–4.2]	109 356	280	2.6 [2.3–2.9]
Slovak Republic	52 522	134	2.6 [2.1–3.0]	52 388	134	2.6 [2.1–3.0]
Slovenia	17 946	100	**5.6 [4.5–6.7]**	17 846	47	2.6 [1.9–3.4]
Spain	456 029	1 438	3.2 [3.0–3.3]	454 591	1199	2.6 [2.5–2.8]
*Sweden*	100 474	316	*3.1 [2.8–3.5]*	100 158	210	2.1 [1.8–2.4]
*UK: England and Wales*	643 407	3 686	***5.7 [5.5–5.9]***	639 721	2185	3.4 [3.3–3.6]
UK: Northern Ireland	22 504	142	**6.3 [5.3–7.3]**	22 362	66	3.0 [2.2–3.7]
UK: Scotland	53 269	358	**6.7 [6.0–7.4]**	52 911	161	3.0 [2.6–3.5]

Cyprus provided no data on fetal death. Ireland only provided data on early neonatal death.

Data for countries that did not adhere to the Euro-Peristat definition are presented in italics.

High mortality rates (>75^th^ quartile) are presented in bold.

### Exclusion of early preterm deaths

**Figure 1:**
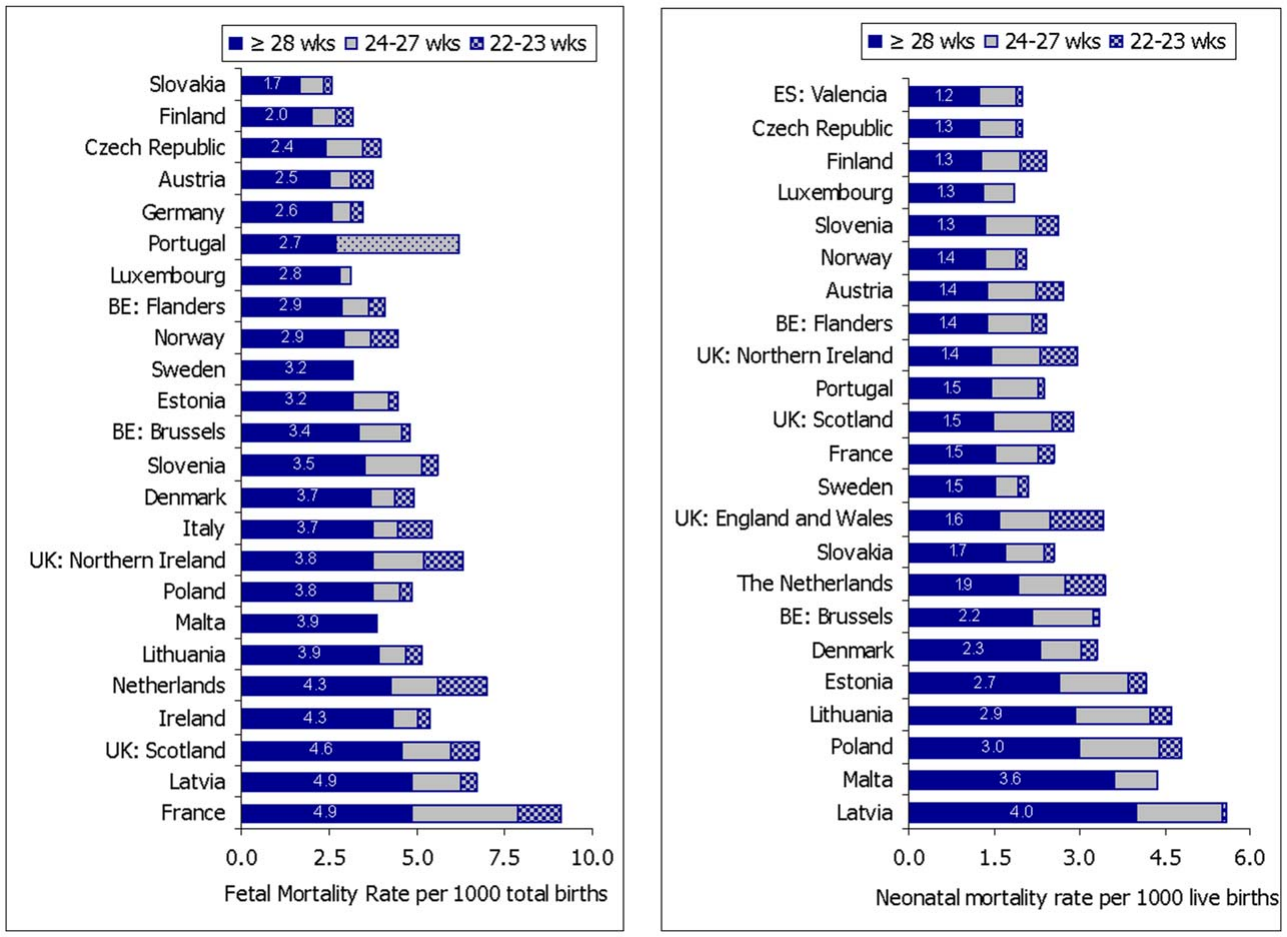
Impact of different inclusion criteria on fetal and neonatal mortality rates. Countries were sorted by mortality rate at or after 28 weeks of gestation with low mortality countries listed first.

Figure 1 illustrates fetal and neonatal mortality rates by gestational-age subgroups and ranks countries by their mortality rate at 28 weeks and over. Excluding early preterm deaths reduced the range of fetal mortality rates quite substantially, while a moderate reduction was observed for neonatal mortality rates. For many countries, deaths before 28 weeks of gestation accounted for a substantial proportion of all deaths. Countries with the highest overall fetal mortality rates did not necessarily have the highest fetal mortality rates at or after 28 weeks of gestation, and not all countries with the highest neonatal mortality rates had the highest neonatal mortality rates at or after 28 weeks of gestation. The fetal mortality rate declined dramatically for France when fetal deaths at 22–27 weeks were excluded and removing neonatal deaths at 22–23 weeks led to a large decline in neonatal mortality rates in the Netherlands, Northern Ireland, and England and Wales.

### Adjusted proportions of fetal and neonatal deaths

**Figure 2:**
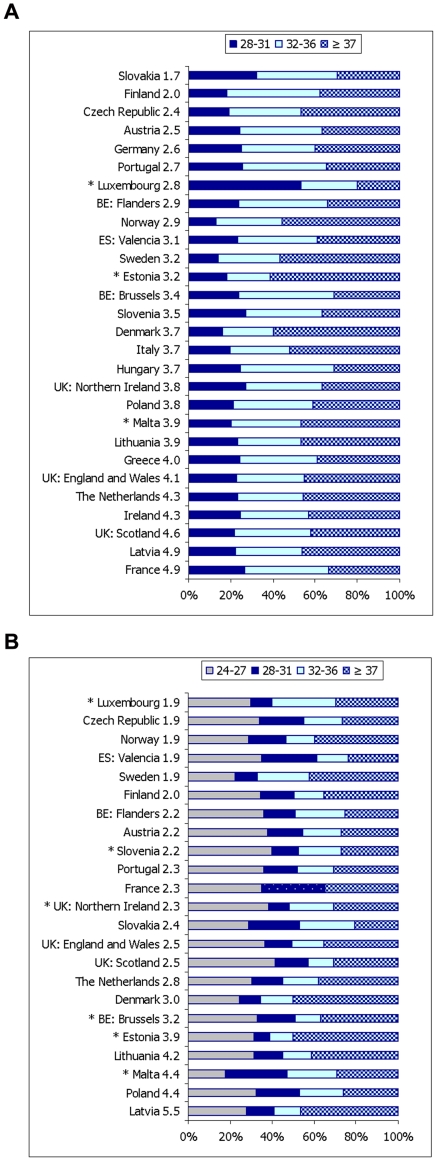
Fetal deaths at or after 28 weeks of gestation (A) and neonatal deaths at or after 24 weeks (B) by gestational age subgroups. Countries were sorted by fetal (A) and neonatal (B) mortality rates with low mortality countries listed first. Fetal mortality rate at or after 28 weeks of gestation was calculated as follows: [(number of fetal deaths ≥28 weeks)/(number of total births ≥28 weeks)]×1000. France, Latvia, Scotland, Ireland and The Netherlands had rates in the top quartile (>75th). * Percentages of fetal deaths were based on fewer than 10 events for Luxembourg and Malta, and for Estonia (at 28–31 and 32–36 weeks). Neonatal mortality rate at or after 24 weeks of gestation was calculated as follows: [(number of neonatal deaths ≥24 weeks)/(number of live births ≥24 weeks)]×1000. Latvia, Poland, Malta, Lithuania, and Estonia had mortality rates in the top quartile (>75th). * Percentages of neonatal death were based on less than 10 events for Luxembourg and Malta, for Brussels, Estonia, and Slovenia (at 28–31 weeks and at 32–36 weeks), and for Northern Ireland (at 28–31 weeks).

Figure 2 presents the percentage of fetal and neonatal deaths in each gestational age subgroup after excluding fetal deaths at 22–27 weeks and neonatal deaths at 22–23 weeks. The percentage of fetal deaths in each gestational age subgroup differed significantly between low and high fetal mortality countries (*p*<0.001). On average, low fetal mortality countries had higher percentages of their fetal deaths at earlier gestational ages (at 28–31 weeks 24.5% vs. 22.9%, at 32–36 weeks 36.3% vs. 32.9%), while high fetal mortality countries had higher percentages at and near term (44.2% vs. 39.2%). In contrast, the percentage of neonatal deaths in each gestational age subgroup did not differ significantly between low and high neonatal mortality countries (*p* = 0.112): at 24–27 weeks (30.0% vs. 31.7%), at 28–31 weeks (17.4% vs. 19.5%), at 32–36 weeks (18.5% vs. 19.6%), and at 37+ weeks (34.1% vs. 29.2%). Indeed, on average, high neonatal mortality countries had lower percentages of neonatal deaths at and near term.

### Gestational age-specific neonatal mortality rates

Table 3 provides data on gestational age-specific neonatal mortality for those countries that could provide both numerator and denominator data for the gestational age distribution. Substantial variation existed within all gestational age subgroups, even when rates based on small denominators were excluded. Countries with high neonatal mortality rates at or after 24 weeks of gestation (Latvia, Poland, Malta, Lithuania, and Estonia) often had the highest gestational age-specific neonatal mortality rates for all gestational age subgroups, except at 22–23 weeks. Some countries stood out in some subgroups. The Netherlands, for instance, had high rates at 22–23 weeks and at 24–27 weeks, while Denmark had high rates at 22–23 weeks and at 37+ weeks.

**Table 3:** Gestational age-specific neonatal mortality rates per 1000 live births.

Country/region	Gestational age in weeks				
	22–23	24–27	28–31	32–36	≥37
Luxembourg	–	–	* 76.9 [0.0–222]	* 9.7 [0.0–20.7]	* 0.6 [0.0–1.3]
Czech Republic	546 [337–754]	218 [170–266]	52.1 [36.2–68.0]	5.9 [3.9–7.9]	0.5 [0.4–0.7]
Norway	556 [326–785]	185 [126–243]	46.6 [26.1–67.0]	4.3 [2.1–6.5]	0.8 [0.6–1.1]
ES: Valencia	–	302 [215–389]	67.4 [41.4–93.5]	3.5 [1.7–5.4]	0.5 [0.3–0.7]
Sweden	485 [314–655]	167 [121–212]	33.4 [19.3–47.4]	8.9 [6.4–11.4]	0.9 [0.7–1.1]
Finland	867 [745–988]	293 [216–371]	52.6 [29.0–76.3]	6.0 [3.0–8.9]	0.7 [0.5–1.0]
BE: Flanders	**1000 [1000–1000]**	311 [237–385]	51.5 [29.5–73.5]	7.2 [4.7–9.8]	0.6 [0.4–0.8]
Austria	867 [767–966]	230 [182–279]	37.4 [24.0–50.7]	4.1 [2.7–5.5]	0.7 [0.5–0.9]
Slovenia	–	308 [182–433]	* 36.5 [5.1–67.9]	* 7.6 [2.4–12.9]	0.7 [0.3–1.1]
Portugal	338 [285–391]	54.9 [38.2–71.7]	6.7 [4.7–8.8]	0.7 [0.6–0.9]	
France	–	–	–	–	0.8 [0.8–0.9]
UK: Northern Ireland	–	244 [151–337]	* 30.3 [4.1–56.5]	9.0 [3.7–14.3]	0.8 [0.4–1.2]
Slovakia	600 [352–848]	281 [203–359]	**86.0 [56.6–115.4]**	**11.8 [7.8–15.8]**	0.5 [0.3–0.7]
UK: England and Wales‡	903 [880–926]	237 [220–254]	36.6 [31.7–41.4]	6.1 [5.3–6.9]	0.9 [0.9–1.0]
UK: Scotland	–	301 [234–367]	47.3 [27.6–67.0]	4.7 [2.4–7.0]	0.8 [0.6–1.1]
The Netherlands	**976 [950–1000]**	**325 [282–368]**	54.5 [42.2–66.7]	7.5 [5.9–9.1]	1.1 [1.0–1.3]
Denmark	**947 [847–1000]**	289 [220–358]	38.2 [21.3–55.0]	8.2 [5.3–11.1]	**1.6 [1.3–2.0]**
BE: Brussels	–	320 [191–449]	* 84.9 [31.8–138]	* 6.6 [1.3–11.8]	1.3 [0.7–1.9]
Estonia	–	**321 [195–446]**	* 47.1 [2.0–92.1]	* 8.8 [1.8–15.8]	**2.1 [1.3–2.8]**
Lithuania	786 [571–1000]	**488 [378–597]**	**90.9 [49.7–132]**	**13.2 [6.9–19.4]**	**1.9 [1.4–2.4]**
Malta	–	**–**	* 238 [55.9–420]	* 15.9 [0.4–31.3]	* 1.4 [0.2–2.6]
Poland	875 [822–928]	**457 [428–486]**	**125 [112–137]**	**16.2 [14.5–17.9]**	1.2 [1.1–1.4]
Latvia	–	**477 [356–598]**	82.9 [42.7–123]	**15.3 [7.3–23.2]**	**2.7 [2.0–3.4]**

Cyprus, Germany, Greece, Hungary, Ireland, and Italy had no data on neonatal death by gestational age. ‡ Data from 2005.

Countries were sorted by neonatal mortality rate at or after 24 weeks of gestation with low mortality countries listed first.

High mortality rates are presented in bold (>75^th^ quartile). Rates based on fewer than 10 deaths were denoted with *.

Rates were not computed for cells with fewer than 10 births and were denoted with –.

For France the number of term live births was estimated from the national perinatal survey and totals from the vital statistics data.

### Correlation between fetal and neonatal mortality rates

**Figure 3:**
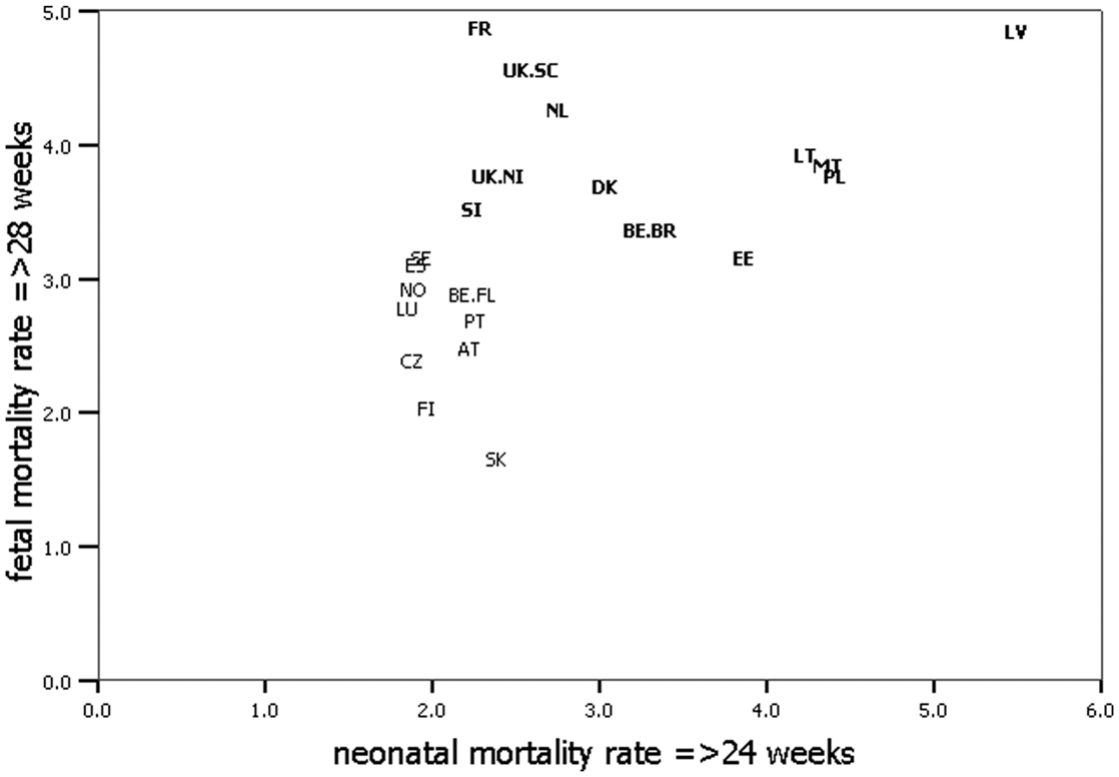
Correlation between fetal and neonatal mortality rates, after exclusion of deaths most likely influenced by registration differences. High mortality countries are presented in bold. Correlation for fetal and neonatal mortality: ρ = 0.646 (*p* = 0.001).

Although fetal mortality rates at or after 28 weeks and neonatal mortality rates at or after 24 weeks were significantly correlated (ρ = 0.646; *p* = 0.001), different patterns were observed for low versus high neonatal mortality countries. Figure 3 shows that high neonatal mortality countries had high fetal mortality rates, but countries with low and moderate neonatal mortality rates had varying levels of fetal mortality. Finland, Czech Republic, Luxembourg, Norway, Spain, and Sweden, for example, all had neonatal mortality rates of 2.0‰, but their fetal mortality ranged from 2.0‰ through 3.5‰. The correlation between fetal and neonatal mortality increased with increasing gestational age and was closest when all preterm deaths were excluded (ρ = 0.758; *p*<0.001), see Figure S1.

### Correlation between early and late neonatal mortality rates

**Figure 4:**
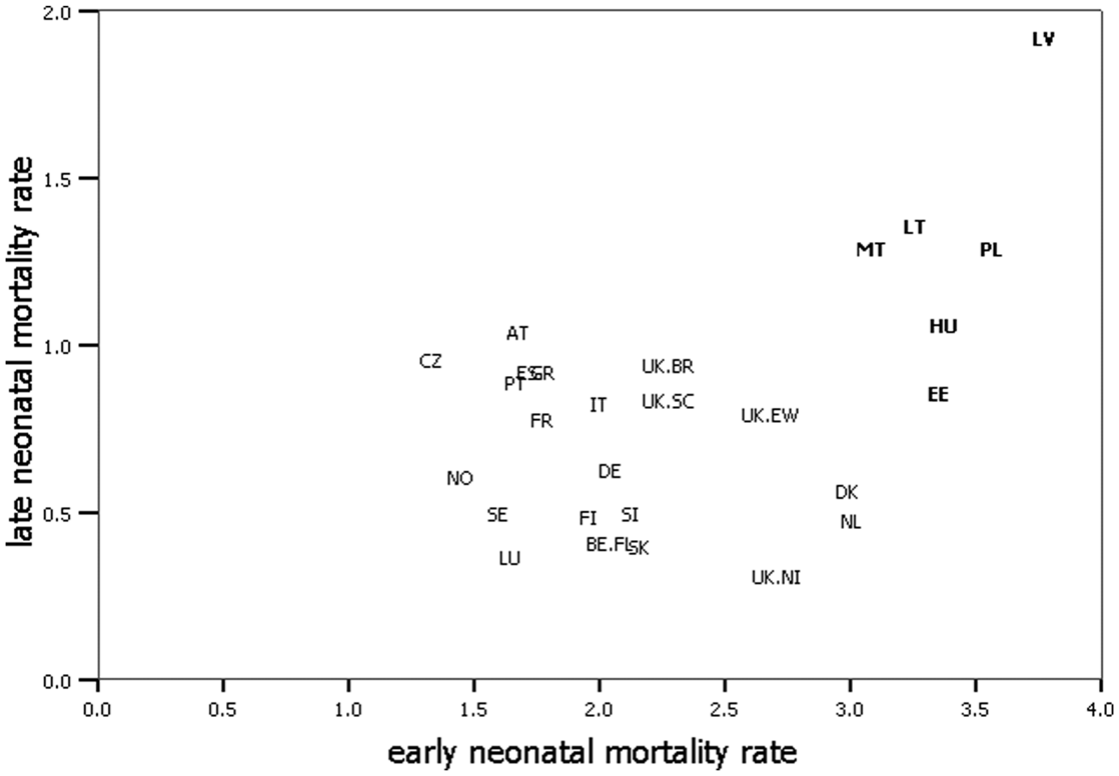
Correlation between early and late neonatal mortality rates. High neonatal mortality countries are presented in bold. ρ = 0.261 (*p* = 0.618) in high neonatal mortality countries versus ρ = −0.302 (*p* = 0.184) in other countries.

Early and late neonatal mortality were related in different ways in countries with high (ρ = 0.261; *p* = 0.618) versus low and moderate (ρ = −0.302; *p* = 0.184) neonatal mortality, as Figure 4 shows. Countries with high neonatal mortality had high rates of both early and late neonatal mortality, while different patterns were observed in other countries: some countries had high early neonatal mortality, but low late neonatal mortality (e.g. the Netherlands and Denmark) and several other countries had low early neonatal mortality and high late neonatal mortality. Early neonatal mortality was related to total neonatal mortality in all countries (ρ = 0.915; *p*<0.001), but late neonatal mortality was related to total neonatal mortality only in countries with high neonatal mortality (ρ = 0.812; *p* = 0.05), see Figure S2.

## Discussion

Our analysis shows that early preterm deaths, most strongly influenced by registration differences, contributed substantially to the variation in fetal and neonatal mortality rates in Europe in 2004. But even after these early preterm deaths were excluded, fetal and neonatal mortality rates varied notably and in all gestational age subgroups, including those at and near term. In addition, patterns of mortality differed for the gestational age at which highest mortality was observed and for the association between fetal and neonatal mortality rates.

Our study has several limitations linked to measurement and data availability, despite efforts within Euro-Peristat to ensure comparability in definitions and sources. The first limitation is related to our ability to assess the completeness of registration of early preterm births, especially stillbirths [11]. Although we were able to describe registration rules, our lack of knowledge about the extent to which these were applied uniformly in all countries limits our ability to assess real fetal mortality at 22–27 weeks and real neonatal mortality at 22–23 weeks. A related issue is the registration of TOP, which is managed very differently from one country to another and terminations are not always included as fetal deaths. TOP varied from 3 to 28% of total fetal deaths in those countries where this proportion could be computed. In France an even larger proportion of early fetal deaths are estimated to be TOP [12]–[13]. To our knowledge, this limitation affects all stillbirth data routinely reported to international agencies. This issue has not previously been highlighted, even though the influence of TOP on fetal mortality rates has been discussed in previous studies [14]–[15].

The different sources of data used by each country may also affect comparability [4], [16]. Some countries provided mortality data from civil or perinatal registries or both, by linkage. These differences in methods may lead to differences in coverage [4]. The number of annual births in each country also varied greatly throughout Europe, from 3902 to 774 870, and random variability from year to year is high when rates are based on small numbers. In Malta, for example, the relatively high gestational age-specific neonatal mortality rate at 28–31 weeks of 238‰ (5 out of 21 babies died) in 2004 fell drastically to 59‰ in the two subsequent years (1 out of 17 babies died in both 2005 and 2006) [17]. In addition, not all countries were able to provide national data; instead, some used either representative samples or regional population-based data. We addressed the question of small sample sizes by calling attention to rates based on fewer than 10 deaths, not reporting those based on fewer than 10 births, and presenting confidence intervals.

A final limitation is related to the comparability of reported gestational ages. Euro-Peristat requested data in completed weeks of gestation based on the best obstetrical estimate available, but we were unable to assess or evaluate differences in the measurement of gestational age. Preterm birth rates may differ depending to whether they are measured by last menstrual period or by ultrasound [18]–[19]. Use of ultrasound measurements, by shifting the entire gestational age distribution to the left, can increase the preterm birth rate [18], but it can also decrease the rate by reducing errors in gestational age estimates [20]. In most European countries, however, dating ultrasounds are now part of standard care during pregnancy, and most women have their first prenatal visit in the first trimester [1].

The Euro-Peristat project chose to collect mortality data using a cut-off point of 22 completed weeks of gestation without a birth weight limit, although data were also collected by birth weight to permit calculation of rates with a lower limit of 1000 grams as recommended by WHO for international comparisons. While the WHO definition of the perinatal period is based on gestational age (starting at 22 weeks of gestation), recommendations for data collection are based primarily on birth weight (death of a fetus that has reached a birth weight of 500 grams or if the birth weight is unavailable, a gestational age of 22 completed weeks or a crown-to-heel length of 24 cm is used) [21]. These international recommendations are understandable because in many countries valid data on gestational age simply do not exist, although it is not clear that stillbirths are systematically weighed [22]. In Europe, however, legislation for registering stillbirths is based largely on gestational age. Regulations governing TOP are also specified with respect to the length of gestation [13].

It is important to note that gestational age-specific mortality data are not produced routinely by any international or European agency (including EUROSTAT, OECD or WHO) and have not, to our knowledge, ever been published in this way from routine data. Our analyses require the collection of fetal and neonatal (both early and late) deaths by individual week of gestation and these are not routinely provided, even in many national publications. A further strength of our analysis is the number of countries that contributed.

From an analytic perspective, gestational-age based analyses are important because they distinguish premature birth from fetal growth restriction. Recent European cohorts of very preterm infants are based on gestational age because of its superior prognostic value [23] and because information on gestational age, and not on birth weight, is available to obstetricians when making decisions during pregnancy and delivery [24]–[27]. Gestational age comparisons across Europe avoid biases related to population differences in birth weight; European comparisons have found that optimum birth weight, defined as the birth weight at which mortality is lowest, varies between European countries [28].

Our analyses showed that it was necessary to exclude stillbirths at 22–27 weeks and neonatal deaths at 22–23 weeks from European comparisons to minimize the effect of differences in registration requirements and TOP legislation on mortality rates. As survival is rare for babies at 22–23 weeks, including this gestational age group adds nearly as many deaths as births and gives this subgroup a large weight in mortality statistics, although they only represent about 1 in 1000 live births. These infants make up a high proportion of fetal and neonatal deaths in some countries. Although registration limits primarily affect fetal deaths, fetal death registration is known to affect the completeness of live birth registration, especially, for example, for those weighing less than 500 grams. The limit of 500 grams primarily concerns infants at 22–23 weeks, so excluding these infants also resolves the problems of comparability presented by birth weight limits. By 24 weeks of gestation, most babies have a birth weight above 500 grams [29]. Some countries had substantially lower gestational age-specific neonatal mortality at 22–23 weeks than reported in specific studies of this population [30]–[31] suggesting that in some countries many immediate neonatal deaths are simply not registered as live births in birth registers. For fetal mortality, a higher cut-off point was necessary to include Sweden, which only registered fetal deaths starting at 28 weeks, as well as to deal with problems of comparability raised by TOP notification. For valid European comparisons of gestation-specific fetal and neonatal mortality, all member states should aim to register fetal deaths from 22 completed weeks of gestation, regardless of birth weight. In July 2008, Sweden changed its limit for registration of stillbirths from 28 to 22 completed weeks of gestation. This change will make it possible to compare their stillbirth rates with other European countries at earlier gestational ages in future studies.

Use of these exclusion criteria had a substantial impact on the ranking of countries. Not all countries with the highest or lowest mortality rates also had the highest or lowest fetal mortality rates at or after 28 weeks or the highest or lowest neonatal mortality rates at or after 24 weeks. It is thus very important for international agencies to collect gestational age to allow like-with-like comparisons. In this light, EUROSTAT's updated directives, which makes provision of fetal and neonatal mortality by birth weight voluntary for EU member states and does not even request voluntary collection by gestational age, is a matter for concern [32].

After exclusion of the early preterm deaths most likely to be influenced by registration differences, the levels and patterns of mortality still varied significantly between countries. The clearest pattern observed was among countries with highest neonatal mortality rates, all new member states of the European Union (Latvia, Poland, Malta, Estonia, Lithuania, and Hungary). These countries had high fetal mortality and high early and high late neonatal mortality rates. This finding suggests that some of the causes may be related to overall standards of living and resources available to the health care system. For Poland and Malta, the restricted availability (Poland) and illegality of TOP (Malta) may also contribute to higher rates.

In contrast, we observed different levels of fetal mortality in other countries with the same overall level of neonatal mortality. Medical advances, such as antenatal steroids and surfactant for very preterm babies, have been very successful in decreasing neonatal mortality, perhaps contributing to more convergent trends in neonatal mortality in countries with similar access to these technologies. In contrast, preventing stillbirths may be more complex. Stillbirths are strongly related to maternal social characteristics, high body mass index and smoking [33]–[34]. The prevalence of these risk factors and health system programs to reduce their impact may differ across countries. Factors suggesting sub-optimal care are associated with a substantial proportion of stillbirths, especially for intrapartum deaths [35].

We also found that some countries had higher mortality in some gestational age subgroups. Denmark, for example, had both high proportions of fetal and neonatal deaths at 37+ weeks, and a high gestational age-specific neonatal mortality rate at 37+ weeks, but was not defined as a high mortality country when comparing rates at earlier gestational ages. France, on the other hand, had the highest fetal mortality rate at or after 28 weeks, but did not have a high proportion of fetal deaths at and near term.

Differences in policies and practices may explain these varying patterns of mortality. For instance, policies related to screening and terminations for congenital anomalies differ in Europe and can have a large impact on both fetal and neonatal mortality rates [5]–[6]. TOP could be performed legally around 2004 in most European countries, although the maximum gestational age limit varied and the notification procedures differed. Exceptions were Ireland, Northern Ireland and Malta, where TOP are not legal and cannot be performed. It is possible that some pregnancies that were found to have lethal congenital anomalies from these countries were terminated elsewhere or that fewer of these pregnancies were terminated; in the latter cases, these babies, who died from the anomaly before, during or after birth, were included in their mortality statistics. Policies related to TOP may explain the relatively high proportion of fetal deaths at and near term in the Netherlands, which, unlike most other European countries in 2004, had no system for systematic early detection of congenital anomalies through prenatal screening. On the other hand, countries that systematically screened at earlier gestational ages and terminated more before the registration limit necessarily have lower mortality rates, both fetal and neonatal [13]–[14]. In contrast, countries that practice terminations at or after 22 weeks may end up with high stillbirth rates explained primarily by terminations. France is one example [13]. Late terminations for fetal anomalies are rare in Europe, and prevalence rates vary between European countries [13]. Ideally, it should be possible to remove TOP from fetal death statistics and to calculate rates with and without TOP.

Another area where European countries differ is in policies and attitudes to withdrawing and withholding care for preterm infants at the limit of viability. This affects the types of care babies receive, their survival probabilities and the timing of death [36]–[37]. Countries that are more likely to withhold care will have higher mortality rates at early gestational ages; for example the Netherlands had high gestational age-specific neonatal mortality rates at 22–23 and at 24–27 weeks, because its active interventions for these early preterm live births were much more limited than in other European countries [36]–[37]. These policies may also affect the proportion of infants that are live born at early gestational ages after medically indicated cesareans [38]. Cesarean delivery rates for infants born between 24 and 25 weeks, excluding cesareans for maternal indications, varied from 0–78% between European regions in 2003, for instance [39]. Without active intervention, neonatal deaths also occur earlier. In the Netherlands, for example, high early neonatal mortality rates but low late neonatal mortality rates could reflect the lack of active intervention before 26 weeks of gestation [36]. Several other countries had low early neonatal mortality rates, but high late neonatal mortality rates, which may indicate that babies were living longer and dying in the late neonatal period.

Finally, high mortality at and near term may reflect policies related to the care of term pregnancies, including policies to induce delivery for post-term infants, which may differ substantially [40] and the organization of maternity services for low-risk pregnancies which also varies greatly within Europe [41]. Having data on causes of death and on timing of death (intrapartum or antepartum) would add to Euro-Peristat's capacity to explain differences in levels and patterns of gestational age-specific mortality and this will be a goal for future phases.

### Conclusions

Registration differences contributed notably to the variability in fetal and neonatal mortality rates between countries. All European countries should use common inclusion criteria for the registration of live and stillbirths. To allow a common analysis of gestation-specific mortality, it is important to have data starting from at least 22 completed weeks of gestation. To comply with WHO recommendations, this would require countries to collect data using both gestational age (22 weeks) and birth weight (500 grams) limits, as is already done in several European countries. Countries should also be able to calculate fetal mortality rates with and without late TOP. Nonetheless, differences in registration criteria do not explain the variability in mortality rates between European countries. Routine reporting of fetal and neonatal deaths by gestational age improves the usefulness of these data for surveillance and policy. Providing countries with international references for neonatal and fetal mortality by gestational age makes it possible for them to assess their specific weaknesses and generate ideas about how to improve outcomes. These data also raise important questions for future research about the tradeoffs between fetal and neonatal mortality in many countries and the reasons for differing gestational age-specific patterns in neonatal mortality.

## Supporting Information

Table S1 **Data sources used for the Euro-Peristat project data.**
(DOC)

Figure S1 **Correlation between gestation-specific fetal and neonatal mortality rates.** Correlation for fetal and neonatal mortality ≥22 weeks: ρ = 0.502 (*p* = 0.010). Correlation for fetal and neonatal mortality ≥28 weeks: ρ = 0.612 (*p* = 0.002). Correlation for fetal and neonatal mortality ≥37 weeks: ρ = 0.758 (*p*<0.001).
(TIF)

Figure S2 **Correlation of early and late neonatal mortality with total neonatal mortality.** High neonatal mortality countries are presented in bold. Correlation for early and total neonatal mortality: ρ = 0.915 (*p*<0.001). Correlation for late and total neonatal mortality: ρ = 0.812 (*p* = 0.05) in high neonatal mortality countries versus ρ = −0.210 (*p* = 0.362) in other countries.
(TIF)
