# Supplementary material for: Gestational Age Patterns of Fetal and Neonatal Mortality in Europe: Results from the Euro-Peristat Project
Source: PLoS One. 2011 Nov 16;6(11):e24727. doi: 10.1371/journal.pone.0024727 (PMC3217927; doi:10.1371/journal.pone.0024727)
Supplement: Table S1 — Data sources used for the Euro-Peristat project data. (DOC) [file pone.0024727.s001.doc]

**Addendum 1. Data sources used for the Euro-Peristat project data**

|  | **Data sources** | **TOP separate system** |
| --- | --- | --- |
| Austria | Statistik Austria (National Birth Statistics) | No |
| BE: Brussels | Linked birth and death certificates | No |
| BE: Flanders | Study Center for Perinatal Epidemiology (SPE) | No |
| Cyprus | Cyprus Statistical Service (CYSTAT),  Vital Statistics | No |
| Czech Republic | Institute of Health Information and Statistics  of Czech republic (UZIS CR) | Abortion Database |
| Denmark | Danish Perinatal Database | National Abortion Registry |
| Estonia | Statistics Estonia, Estonian Medical Birth Registry, and Estonian Mortality Database | Estonian Abortion Registry |
| Finland | Medical Birth Register | Register on Induced Abortions |
| France | National Perinatal Survey | No |
| Germany | www.bqs-online.de | [www.destatis.de](http://www.destatis.de/) |
| Greece | National database | No |
| Hungary | Hungarian Central Statistics Office | Yes |
| Ireland | National Perinatal Reporting System (NPRS) | No |
| Italy | National Birth Certificates Register | National ongoing survey on induced abortions  - Istat - National Institute of Statistics - |
| Latvia | Newborns Register of Latvia  and Death Cause Data Base | Routine statistics |
| Lithuania | Medical Data of Births | Lithuanian Health Information Center  (LHIC), annual report data |
| Luxembourg | FIMENA Fiche Médicale de Naissance and Mortality statistics / Ministry of health | No |
| Malta | National Obstetrics Information System (NOIS) National Mortality Register | TOP is illegal and not performed |
| The Netherlands | The Netherlands Perinatal Registry | Register on Induced Abortions |
| Norway | Medical Birth Registry of Norway | Termination of Pregnancy Register run by and under  the Regulations of the Medical Birth Registry of Norway |
| Poland | Birth and death certificates | Ministry of Health |
| Portugal | Demographic Statistics -  National Institute of Statistics | No |
| Slovakia | Správa o novorodencovi Z (MZ SR) 5-12  (Report on newborn - Z (MZ SR) 5-12) | Report on spontaneous abortion and  interruption of pregnancy - Z (MZ SR) 7-12 |
| Slovenia | National perinatal system of Slovenia | Fetal deaths database |
| Spain | National Institute for Statistics (INE); Perinatal Mortality Register (region Valencia) | Induced Abortions Registry (national) |
| Sweden | Medical Birth Register | Swedish birth defects registry |
| UK: England and Wales | Civil registration of births and deaths | Abortion notifications cover all TOP in England and Wales, including residents of Northern Ireland and Ireland |
| UK: Northern Ireland | The Confidential Enquiry into Maternal  and Child Health (CEMACH) | No |
| UK: Scotland | Scottish Stillbirth and Infant Death Enquiry | Abortion Act Statistics  (Notifications to the Chief Medical Officer for Scotland of abortions performed under the Abortion Act 1967) |
