# Supplementary figures and images for: Gestational Age Patterns of Fetal and Neonatal Mortality in Europe: Results from the Euro-Peristat Project
Source: PLoS One. 2011 Nov 16;6(11):e24727. doi: 10.1371/journal.pone.0024727 (PMC3217927; doi:10.1371/journal.pone.0024727)

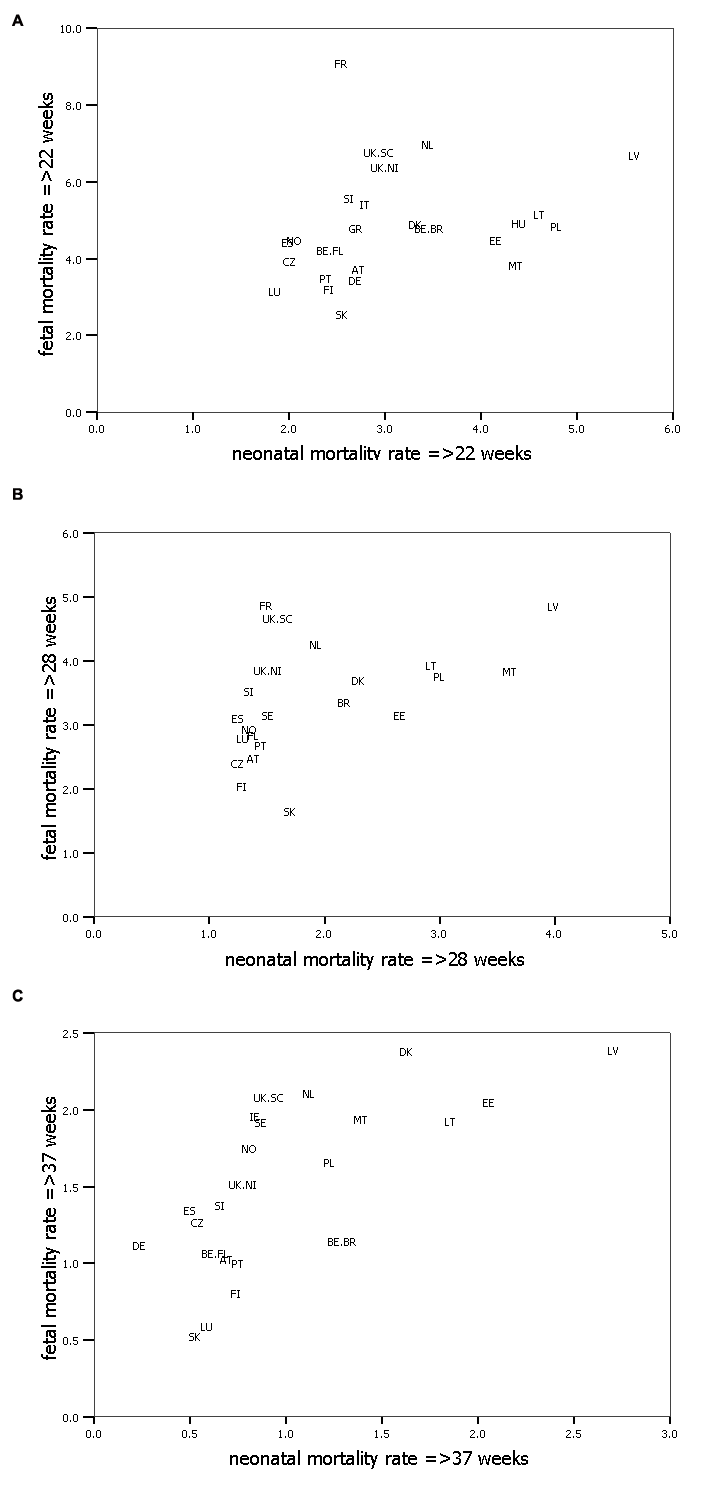

Supplement: Figure S1 — Correlation between gestation-specific fetal and neonatal mortality rates. Correlation for fetal and neonatal mortality ≥22 weeks: ρ = 0.502 (p = 0.010). Correlation for fetal and neonatal mortality ≥28 weeks: ρ = 0.612 (p = 0.002). Correlation for fetal and neonatal mortality ≥37 weeks: ρ = 0.758 (p<0.001). (TIF) [file pone.0024727.s002.tif]

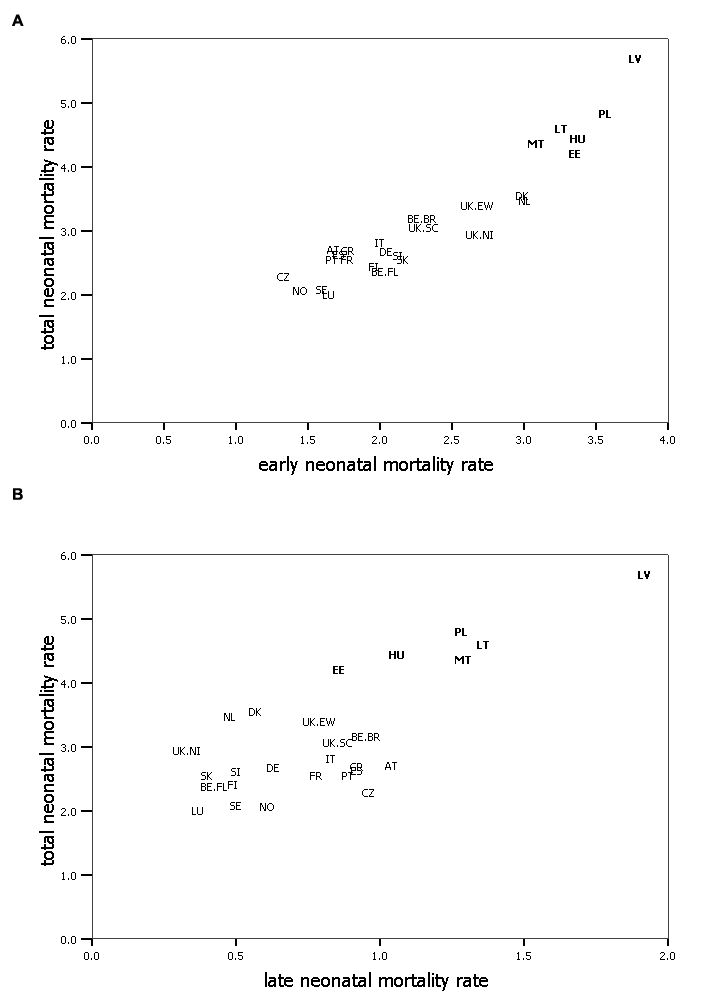

Supplement: Figure S2 — Correlation of early and late neonatal mortality with total neonatal mortality. High neonatal mortality countries are presented in bold. Correlation for early and total neonatal mortality: ρ = 0.915 (p<0.001). Correlation for late and total neonatal mortality: ρ = 0.812 (p = 0.05) in high neonatal mortality countries versus ρ = −0.210 (p = 0.362) in other countries. (TIF) [file pone.0024727.s003.tif]
